# Integrated quality evaluation strategy for multi-species resourced herb medicine of Qinjiao by metabolomics analysis and genetic comparation

**DOI:** 10.1186/s13020-020-0292-3

**Published:** 2020-02-11

**Authors:** Zeyun Li, Yue Du, Yongliang Yuan, Xiaojian Zhang, Zhengtao Wang, Xin Tian

**Affiliations:** 1 Department of Pharmacy, The First Affiliated Hospital of Zhengzhou University, Zhengzhou, 450052 China; 2 Institute of Chinese Materia Medica, Shanghai University of Traditional Chinese Medicine, Shanghai, 201203 China

**Keywords:** Quality evaluation, Multi-species resourced herb medicine (MSRHM), Qinjiao, ITS2, Metabolomics

## Abstract

**Background:**

Quality evaluation of multi-species resourced herb medicine (MSRHM) is a main problem for quality control of herb medicine. Current quality evaluation methodology lost consideration of species discrepancy. New quality evaluation strategy for MSRHM is in urgent need. Qinjiao, a representative MSRHM, originated from *Gentiana macrophylla* Pall., *Gentiana straminea* Maxim., *Gentiana crassicaulis* Duthie ex Burk. or *Gentiana dahurica* Fisch., has been used as an important herb medicine over 2000 years for expelling wind-dampness and relieving impediment pain. However, quality evaluation among species has never been revealed. The current work proposes an integrated quality evaluation strategy for MSRHM of Qinjiao, which may promote innovation of quality control of MSRHM.

**Methods:**

In this work, 58 batches of Qinjiao covering 4 species were collected. Genetic comparative analysis based on ITS2 sequence was conducted. Metabolomics analysis based on TOF–MS and NMR spectrum were carried out. Compounds underlying species differences were identified and their discrepancies among species were investigated by ANOVA analysis and multivariate analysis.

**Results:**

Four species of Qinjiao can be authenticated by ITS2 sequence comparation. Metabolomics analysis by TOF/MS and NMR revealed chemical discrepancies among species of Qinjiao. Maximum discrepancy was present between *Gentiana crassicaulis* Duthie ex Burk. and *Gentiana dahurica* Fisch. Chemical difference among species were tentative explored. For TOF–MS profiling, 28 constituents were tentative identified, 17 of which were further confirmed by standards. For ^1^H-NMR profiling, signals from 5 compounds were assigned. Contents discrepancies were investigated by ANOVA analysis. It seems that (seco)iridoids like loganic acid, gentiopicroside or swertiamarin were richer in specie of *Gentiana crassicaulis* Duthie ex Burk., while flavonoid (morroniside) and triterpenoids (roburic aicd, ursolic acid, oleanolic acid, β-sitosterone) were richer in specie of *Gentiana dahurica* Fisch. The current research demonstrates that metabolite profiling based on both UPLC/Q-TOF MS and ^1^H-NMR coupled with ITS2 sequence comparation can be a powerful tool for quality investigation of MSRHM of Qinjiao.

**Conclusions:**

A comprehensive quality evaluation strategy for MSRHM was proposed by integrating UPLC-Q-TOF–MS, NMR based metabolic analysis and ITS2 sequence genetic comparation. The proposed quality evaluation strategy shall promote innovation of quality control of traditional Chinese medicine.

## Background

Quality control of multi-species resourced herb medicine (MSRHM) is a main problem for quality control of herb medicine. Current quality control methodology bears disadvantages of lacking consideration of species discrepancy, ignoring the fact that discrepancies among species were inevitable and shall produce different chemical component and clinical effects. As a result, new quality investigation strategy for MSRHM is in urgent need.

Qinjiao, namely *Gentianae Macrophyllae* Radix, is an ancient Chinese herb medicine and has been described and recorded in several ancient Chinese Medicine monographs like Shen Nong’s Herbal Classic (Han Dynasty, Shen Nong Ben Cao Jing), Compendium of Materia Medica (Ming Dynasty, Ben Cao Gang Mu) [1] and also in Chinese pharmacopoeia. Over 2000 years, Qinjiao has been utilized to treat a wide range of diseases, including hypertension, osteoarthritis, and especially rheumatism [2]. According to China Pharmacopoeia 2015 version [3], Qinjiao consists of the dried roots of *Gentiana macrophylla* Pall. (*G. macrophylla*), *Gentiana straminea* Maxim. (*G. straminea*), *Gentiana crassicaulis* Duthie ex Burk. (*G. crasicaulis*) or *Gentiana dahurica* Fisch. (*G. daurica*). As a represented MSRHM, quality and efficacy of Qinjiao among species were inevitable variable [4, 5]. Former researches mainly focused on contents determination or chemical profiling of certain kind of Qinjiao [6, 7], or even chemical and genetic analysis of *G. crasicaulis* and *G. macrophylla* [8]. No systematic species investigation for four kinds of Qinjiao has been revealed, which may shed new light into quality control and clinical utilization of Qinjiao.

DNA barcode of internal transcribed spacer 2 (ITS2) is prevalently adopted as a universal barcode for plant, especially herbal medicinal identification [9]. ITS2 barcode has been successfully employed for species identification of Qinjiao [8, 10]. Chemical profiling combined with multivariate analysis provided systematic chemical comparison of metabolites, and can be powerful tool for species investigation of MSRHM [11]. Ultra-performance liquid chromatography quadrupole time-of-flight mass spectrometry (UPLC-Q-TOF–MS) and proton nuclear magnetic resonance spectrometer (^1^H-NMR) are the most frequently employed platform for metabolic profiling.

**Fig. 1:**
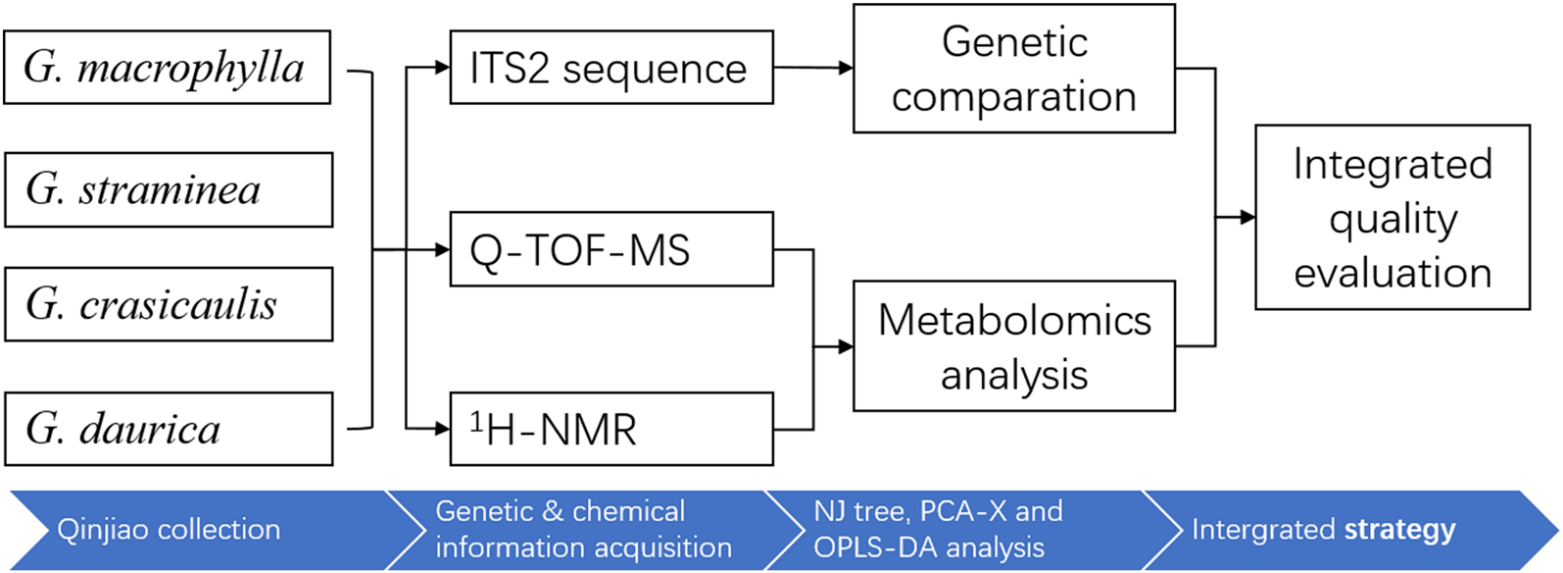
The flow chart of the proposed methodology

Herein, this research collected 58 batches of Qinjiao, covering four species, originated from main producing areas of China (Gansu, Shanxi, Hebei, Shaanxi, Heilongjiang, Liaoning, Nei Mongol, Yunnan, Szuchuan, Qinghai, Tibet). Genetic comparative and metabolic profiling analysis of four species of Qinjiao were carried out. First, all herbs were authenticated by morphological identification as well as DNA barcoding comparison. Then, chemical profiles were acquired by UPLC-Q-TOF–MS and ^1^H-NMR. Third, metabolomics based species investigation, including principal component analysis (PCA) and orthogonal partial least squares discrimination analysis (OPLS-DA), was conducted to reveal the quality discrepancy among species. Finally, an integrated quality evaluation strategy for MSRHM of Qinjiao by UPLC-Q-TOF–MS, NMR based metabolomics analysis and ITS2 sequence genetic comparation was established. The flow chart of the proposed methodology is shown in Fig. 1. The current work may facilitate quality control, utilization and species discrimination of different kinds of Qinjiao.

## Materials and methods

### Sample collection and preparation

Fifty-eight batches of rhizomes of *G. macrophylla*, *G. straminea*, *G. crasicaulis* and *G. daurica* were collected from different herbal markets or harvested from various locations of China. All samples were authenticated by Professor Jiuzhi Yuan (Shenyang Pharmaceutical University) or Professor Lihong Wu (Shanghai University of Traditional Chinese Medicine). Voucher specimens were deposited in Department of Pharmacy, The First Affiliated Hospital of Zhengzhou University, Zhengzhou, China or the Institute of Chinese Materia Medica, Shanghai University of Traditional Chinese Medicine, Shanghai, China. Detailed information of collected samples in this study is list in Table 1. The roots were gently washed and dried at 50 °C for 48 h, and then were grounded into powder and stored in glass jars in the dark at room temperature until further analysis.

**Table 1:** Samples information of Qinjiao for metabolomics and ITS2 barcode analysis

Sample ID	Origins	Species	GenBank accession nos.
QJ01	Mongolian autonomous county of Henan, Tibetan autonomous prefecture of Huangnan, Qinghai province	*G. macrophylla* Pall.	MH602351
QJ02	Gonjo county, Qamdo city, Tibet	*G. crassicaulis* Duthie ex Burk.	MH602352
QJ03	Tibetan Qiang autonomous prefecture of Ngawau, Sichuan	*G. crassicaulis* Duthie ex Burk.	MH602353
QJ04	Nyingchi city, Tibet autonomous region	*G. crassicaulis* Duthie ex Burk.	MH602354
QJ05	Ganzi Tibetan autonomous prefecture, Sichuan province	*G. crassicaulis* Duthie ex Burk.	MH602355
QJ06	Maqu country, Gannan Tibetan autonomous prefecture, Gansua province	*G. straminea* Maxim.	MH602389
QJ07	Tibetan autonomous prefecture of Golog, Qinghai province	*G. straminea* Maxim.	MH602390
QJ08	Mongolian autonomous county of Henan, Tibetan autonomous prefecture of Huangnan, Qinghai province	*G. straminea* Maxim.	MH602391
QJ09	Ebian Yi Nationality autonomous county, Minle city, Sichuan province	*G. crassicaulis* Duthie ex Burk.	MH602356
QJ10	Hohhot city, Inner Mongolia	*G. straminea* Maxim.	MH602392
QJ11	Daguan country, Zhaotong city, Yunnan province	*G. crassicaulis* Duthie ex Burk.	MH602357
QJ13	Yulong naxi autonomous prefecture, Lijiang city, Yunan province	*G. crassicaulis* Duthie ex Burk.	MH602358
QJ14	Minle country, Zhangye city, Gansu provinice	*G. straminea* Maxim.	MH602393
QJ15	Xinzhou city, Shanxi province	*G. crassicaulis* Duthie ex Burk.	MH602359
QJ16	Gonghe town, Qingyang city, Gansu province	*G. macrophylla* Pall.	MH602387
QJ17	Barkam city, Tibetan Qiang autonomous prefecture of Ngawau, Sichuan	*G. crassicaulis* Duthie ex Burk.	MH602360
QJ18	Barkam city, Tibetan Qiang autonomous prefecture of Ngawau, Sichuan	*G. crassicaulis* Duthie ex Burk.	MH602361
QJ19	Barkam city, Tibetan Qiang autonomous prefecture of Ngawau, Sichuan	*G. crassicaulis* Duthie ex Burk.	MH602362
QJ20	Ruoergai County, Tibetan Qiang Autonomous Prefecture of Ngawau, Sichuan	*G. straminea* Maxim.	MH602394
QJ21	Ruoergai county, Tibetan Qiang autonomous prefecture of Ngawau, Sichuan	*G. straminea* Maxim.	MH602395
QJ22	Huating country, Pinliang city, Gansu province	*G. macrophylla* Pall.	MH602388
QJ23	Tongguan country, Weinan city, Shanxi province	*G. crassicaulis* Duthie ex Burk.	MH602363
QJ24	Malong country, Qujing city, Yunana province	*G. crassicaulis* Duthie ex Burk.	MH602364
QJ25	Huichuan town, Weiyuan country, Dingxi city, Gansu province	*G. crassicaulis* Duthie ex Burk.	MH602365
QJ26	Ludian country, Zhaotong city, Yunnan province	*G. crassicaulis* Duthie ex Burk.	MH602366
QJ27	Tibetan Qiang autonomous prefecture of Ngawau, Sichuan	*G. dahurica* Fisch.	MH602405
QJ28	Guizhou province	*G. crassicaulis* Duthie ex Burk.	MH602367
QJ29	Yulong naxi autonomous prefecture, Lijiang city, Yunan province	*G. crassicaulis* Duthie ex Burk.	MH602368
QJ30	Heishui country, Tibetan Qiang Autonomous prefecture of Ngawau, Sichuan	*G. crassicaulis* Duthie ex Burk.	MH602369
QJ31	Heishui country, Tibetan Qiang autonomous prefecture of Ngawau, Sichuan	*G. straminea* Maxim.	MH602396
QJ32	Jingping country, Suzhou city, shanxi province	*G. dahurica* Fisch.	MH602406
QJ33	Weixi Lisu autonomous county, Diqing Tibetan autonomous Prefecture, Yunan province	*G. crassicaulis* Duthie ex Burk.	MH602370
QJ34	Keshiketeng Banner, Linxi County, Neimenggu province	*G. straminea* Maxim.	MH602397
QJ35	Nima town, Maqu country, Gannan Tibetan autonomous prefecture, Gansu province	*G. straminea* Maxim.	MH602398
QJ36	Tibetan Qiang autonomous prefecture of Ngawau, Sichuan	*G. straminea* Maxim.	MH602399
QJ37	Tibet autonomous region	*G. crassicaulis* Duthie ex Burk.	MH602371
QJ38	Ping’an country, Haidong city, Qinghai province	*G. straminea* Maxim.	MH602400
QJ39	Yunan province	*G. crassicaulis* Duthie ex Burk.	MH602372
QJ40	Yulong naxi autonomous prefecture, Lijiang city, Yunan province	*G. crassicaulis* Duthie ex Burk.	MH602373
QJ41	Dali Bai autonomous prefecture, Yunan province	*G. crassicaulis* Duthie ex Burk.	MH602374
QJ42	Tibet autonomous region	*G. crassicaulis* Duthie ex Burk.	MH602375
S0	Tu autonomous county of Huzhu, Qinghai province	*G. dahurica* Fisch.	MH602402
S1	Yunan province	*G. crassicaulis* Duthie ex Burk.	MH602377
S2	kunming city, Yunna province	*G. crassicaulis* Duthie ex Burk.	MH602378
S3	kunming city, Yunna province	*G. crassicaulis* Duthie ex Burk.	MH602379
S4	Yunan province	*G. crassicaulis* Duthie ex Burk.	MH602380
S5	Lijiang city, Yunan province	*G. crassicaulis* Duthie ex Burk.	MH602381
S6	Yunan province	*G. crassicaulis* Duthie ex Burk.	MH602382
S7	Tibetan autonomous county of Muli, Sichuan province	*G. crassicaulis* Duthie ex Burk.	MH602383
S8	Yunan province	*G. crassicaulis* Duthie ex Burk.	MH602376
S9	Yunan province	*G. macrophylla* Pall.	MH602384
S10	Unknown	*G. straminea* Maxim.	MH602401
S11	Gansu province	*G. macrophylla* Pall.	MH602385
S12	Nei Monggol autonomous region	*G. dahurica* Fisch.	MH602407
S13	Zhangjiakou city, Hebei province	*G. dahurica* Fisch.	MH602408
S14	Helongjiang province	*G. dahurica* Fisch.	MH602403
S16	Nei Monggol autonomous region	*G. dahurica* Fisch.	MH602404
S18	Gansu province	*G. macrophylla* Pall.	MH602386

QJ01–QJ42 were deposited in department of pharmacy, the first affiliated hospital of Zhengzhou University, Zhengzhou, China; S0–S18 were deposited the Institute of Chinese Materia Medica, Shanghai University of Traditional Chinese Medicine, Shanghai, China

### Chemicals and reagents

Methanol-d4 (CD3OD, 99.8%) was obtained from Cambridge Isotope Laboratories (Miami, FL, USA). Methanol of HPLC grade was purchased from Honeywell Inc. (Morristown, NJ, USA). Formic acid of LC–MS grade was bought from ROE Scientific Inc. (Newark, DE, USA). Deionized water was purified using a Milli-Q system (Millipore, Bedford, MA, USA). The chemical reference standards (CRS) of gentiopicroside, loganic acid, swertiamarin, sweroside, 6′-*O*-β-d-Glucosylgentiopicroside, roburic acid, morroniside, isovitexin, homoorientin, oleanolic acid, ursolic acid, β-sitosterone, citric acid, quercetin, kaempferol, and daucosterol were purchased from Chengdu Must Bio-techenology Co., Ltd. (Chengdu, China), with HPLC purity > 98%. All other chemicals and reagents were of analytical grade and commercially available.

### Genetic Analysis of collected Qinjiao samples

ITS2 was prevalently adopted as a universal barcode for plant, especially herbal medicinal identification [9]. ITS2 barcode has been successfully employed for species identification of Qinjiao [8, 10]. In current research, ITS2 region was compared to verify the authentication of collected Qinjiao samples. To extract total genomic DNA from dried rhizomes (50 mg), the protocol provided by the Plant Genomic DNA Kit (Tiangen Biotech, Co., Ltd., Beijing, China) was used. Extracted DNA sample was stored at − 20 °C until use. The ITS2 sequence was amplified using a pair of primers (ITS2F: 5′-ATGCGATACTTGGTGTGAAT-3′; ITS3R: 5′-GACGCTTCTCCAGACTACAAT-3′) described previously by Chen et al. [12]. The PCR amplification was conducted as described by Luo et al. [13]. Amplification products were examined by electrophoresis in 1% (wt/vol.) agarose gels and visualized under ultraviolet light to detect successfully amplified products and the possible contamination of negative controls. After purifying, the PCR products were directly subjected to sequencing.

Sequences were edited and assembled using DNAMAN software (version 6.0) and refined manually. ITS2 resuquence were identified using DNA barcoding system for identifying herbal medicine (http://www.tcmbarcode.cn). Genetic distances were calculated using the Kimura-2-Parameter (K2P) model. All the newly obtained ITS2 sequences were uploaded to GenBank.

### UPLC-Q-TOF–MS profiles analysis of the collected Qinjiao

0.5 g power (through No. 3 sieve) of each Qinjiao sample was placed into a separate 50 mL stopper conical flask followed by the addition of 20 mL methanol. The mixtures were vortexed for 1 min, sonicated (40 kHz, 500 W) for 30 min. Cooling down to ambient temperature, the lost weight was made up by methanol. After centrifugation at 10,000 rpm, 4 °C for 10 min, a 2 μL aliquot of the supernatants was injected into a UPLC-ESI-Q-TOF system (AB Sciex, Framinghan, MA, USA) for MS analysis.

Chromatography separation was achieved on an ACQUITY UPLC HSS T3 column (100 × 2.1 mm i.d., 1.8 μm) maintained at 40 °C. The mobile phase consisted of A (0.1% formic acid in water) and B (0.1% formic acid in methanol), using gradient elution: 0–1 min, 5–30% B; 1–5 min, 30–40% B; 5–6 min, 40–90% B; 6–13 min, 90–100% B; 13–21 min, 100% B; 21.01–24 min, 5% B. The flow rate was set at 0.3 mL/min and the injection volume was 2 μL.

The mass spectrometric data were collected on a SCIEX X500R QTOF mass spectrometer (AB Sciex, Framinghan, MA, USA*)* coupled with an electrospray ionization interface in negative ion modes (ESI-). SCIEX OS software 1.2 (AB, Milford, MA) was employed for data acquisition and procession. The following parameters settings were used: the ion spray voltage of 4000 V; turbo spray temperature (TEM) of 600 °C; declustering potential (DP) of − 80 V; collision energy (CE) of − 45 V; nebulizer gas (gas 1) of 55 psi; heater gas (gas 2) of 55 psi, CAD gas of 7 psi, and curtain gas of 35. Nitrogen was kept as the nebulizer and auxiliary gas. TOF MS and TOF MS/MS were scanned with the mass range of *m/z* 50–1000. Continuous recalibration was carried out every six samples. In addition, dynamic background subtraction (DBS) trigger information-dependent acquisition (IDA) was used to trigger acquisition of MS/MS information of low-level constituents. The accurate mass and composition for the precursor ions and fragment ions were analyzed using the Markerview™ software (Version 4.1, Waters Co., Milford, MA, USA) integrated with the instrument.

QC samples were prepared by combining equal aliquots from all Qinjiao samples and were injected every six specimens during the whole analysis. QC data obtained was used to assess the stability of the LC/MS platform. For all QCs, 5 characteristic features (list in Additional file 1: Table S1) were picked out to verify the stability. The results proved that variations of retention times were less than 0.2 min, drift values of *m/z* were less than 10 PPM, and the RSD of peak areas were all below 10% (Additional file 1: Table S1).

Raw data from Q/TOF–MS were analyzed using Markerview for peak deconvolution and peak alignment with the following parameters: initial retention time 0.5 min, final retention time 23 min, mass tolerance 10 PPM, ion intensity threshold (3000 counts) and retention time tolerance 0.1 min. The data were combined into a single matrix by aligning peaks with the same mass-retention time pair together from each data file in the data set. The ion intensities of each peak detected (2806 MS features for ESI- modes) were normalized to the sum of the peak intensities in each sample. After normalization, the data was processed according to the “80% rule”, briefly only variables with values above zero presenting in at least 80% of each group were kept for the following analysis [14].

## ^1^H-NMR profiles analysis of the collected Qinjiao

The methanol extracting supernatants (600 μL) were dried down using a centrifugal vacuum concentrator and were redissolved in 600 μL of MeOD. After mixing well, 450 μL of reconstitution wan transferred into the 5 mm NMR tubes (Norell, Landisville, NJ, USA) for NMR analysis.

NMR spectral data were obtained at 300 K on a Bruker 600-MHz AVANCE III NMR spectrometer (Bruker, Germany), equipped with a 5.0-mm BBO probe, operating at 600.13 MHz for ^1^H. The zg30 Bruker pulse program was used for 1D ^1^H NMR, with a TD of 64 k, relaxation delay of 1 s, spectral width of 20 ppm, and 256 scans. A line-broadening factor of 0.3 Hz was applied to FIDs before Fourier transformation. All NMR spectra were phased and baseline-corrected manually using TOPSPIN 3.5 (Bruker, Germany). The spectra were referenced internally to the chemical shift of H-3 signal of gentiopicroside at 7.46 ppm. Each ^1^H-NMR spectrum over the ranged 0.5–10.0 ppm was reduced to 238 regions of equal width (0.04 ppm) and the signal intensity in each region was integrated using AMIX (version 3.9, Bruker, Germany). The region of 4.75–5.20 ppm was removed prior to any statistical analysis in order to eliminate any residual water signal. Then data was normalized in AMIX by dividing each integrated segment by the total area of the spectrum to reduce any significant.

### Statistical analysis and compound annotation

Output data from TOF–MS or NMR analysis was separately imported into SIMCA (version 14.0, Umetrics, Umeå, Sweden) for multivariate statistical analysis (MS data unit variance scaled, NMR data pareto-scaled). To provide comparative interpretations and visualization of the metabolic differences among the four species of Qinjiao, PCA and OPLS-DA were applied to the TOF–MS or NMR data set. The quality of the models was described by R2X and Q2 values. R2X shows the proportion of variance in the data explained by the models and indicates goodness of fit. The value closer to 1 indicates the goodness of fit. Q2, on the other hand, shows the proportion of variance in the data predictable by the model and indicates predictability. The results were visualized in the form of score plots, where each point represents an individual sample (to show the group clusters), and loading plots or S-plots, where each coordinate represents one mass-retention feature or ^1^H-NMR spectral region (to identify the variables contributing to the classification). The variable importance of projection (VIP) is the vector to summarize the total importance of the variable in explaining the model. The corresponding variables with VIP > 1.0 were chosen as potential discriminative metabolites. To justify the OPLS-DS models, analysis of variance testing of Cross-Validated predictive residuals (CV-ANOVA) were conducted. CV-ANOVA is a diagnostic tool for assessing the reliability of PLS and OPLS models. The *P* value produced by CV-ANOVA indicates the probability level. The common practice is to interpret a p-value lower than 0.05 as pointing to a significant model. Statistical analysis was also performed using one-way analysis of variance (ANOVA) followed by Tukey’s multiple comparison test (SPSS, Chicago, IL, USA). A probability of P < 0.05 was considered to be statistically significant between two groups.

LC–MS Peaks were identified according to actual mass, MS/MS fragments and retention time (RT). First, the m/z value of the molecular ion of interest was searched against a self-build Qinjiao constituent Database, where data was collected from published researches [15, 16]. Then, the putative identifications were verified by comparing the MS2 fragmentations. Part of the constituents were further identified by reference standards. ^1^H-NMR signals were assigned by comparing the spectrum of Qinjiao with that of gentiopicroside, loganic acid, sweroside, swertiamarin or sucrose by Chenomx NMR suit (version7.6, Chenomx, Edmonton, Canada) with method described in our previous work [17].

## Results

### ITS2 comparation

**Fig. 2:**
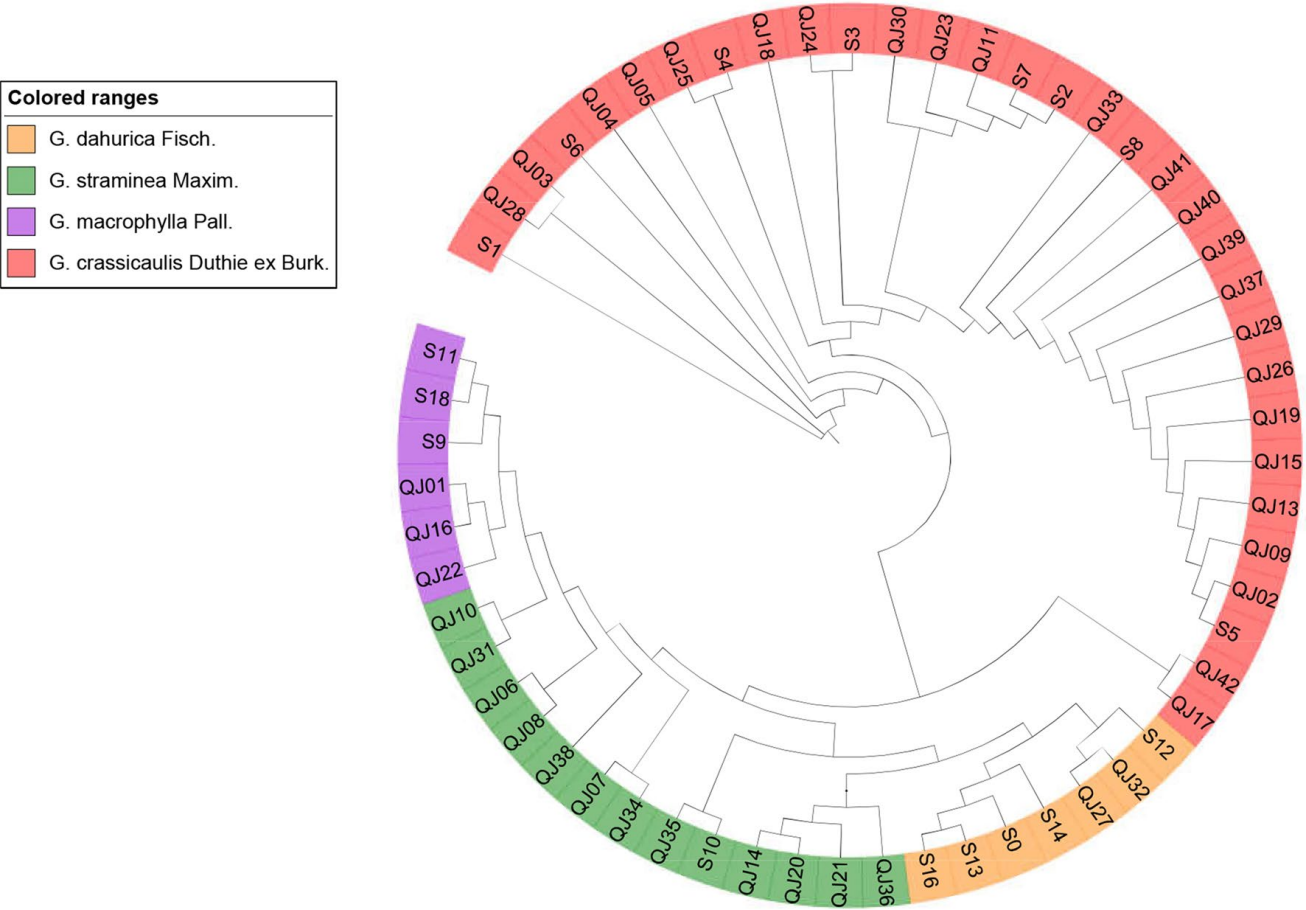
NJ tree of *G. macrophμlla*, *G. straminea*, *G. crassicaulis* and *G. dahurica*

ITS2 sequences from collected Qinjiao samples were submitted to GenBank database (Accession numbers were listed in Table 1), assembled with CodonCode Aligner 3.7.1 (CodonCode Co., Dedham, MA, USA) and aligned using ClustalW. Kimura 2-Parameter (K2P) distances. GC content of base and NJ trees were calculated and constructed using the MEGA X software with the Bootstrap method (500 resampling) and K2P model [18]. The distances within or among species are separately list in Table 2. The average ITS2 region was 497 bp in length in 4 species of Qinjiao, and the G+C content ranged from 55.7% to 56.2%, with an average of 56.0% (Table 2). A neighbor-joining (NJ) tree of ITS2 barcode was formed on the basis of K2P model (Fig. 2).

**Table 2:** Sequence sizes, percent G+C content and mean distance intra/inter each spices of collected Qinjiao samples

Species	ITS2 length (bp)	G+C content (%)	Mean distance intra species	Mean distance inter species		
				1	2	3
1 *G. crassicaulis* Duthie ex Burk.	495.8	56.0	0.0274			
2 *G. dahurica* Fisch.	496.0	55.9	0.0229	0.0371		
3 *G. macrophylla* Pall.	498.2	55.7	0.0062	0.0312	0.0246	
4 *G. straminea* Maxim.	498.8	56.2	0.0130	0.0288	0.0220	0.0146

### TOF–MS data metabolomics

**Fig. 3:**
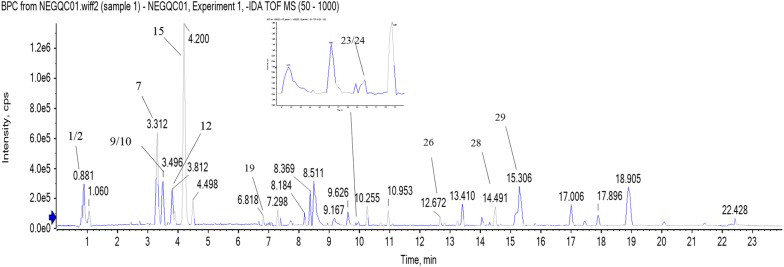
Representative BPI chromatograms of *G. crassicaulis*, with part of the constituents identified. Note: 1. Sucrose, 2. Swertiajaposide A, 7. Loganic acid, 9. 6′-*O*-β-d-Glucosylgentiopicroside, 10. Qinjiaoside A, 12. Swertiamarin, 15. Gentimacroside, 19.Vitexin, 23. Ursolic Acid, 24. Oleanolic acid, 26. 2α-hydroxyl ursolic acid, 28. β-sitosterone, 29. Roburic acid

**Fig. 4:**
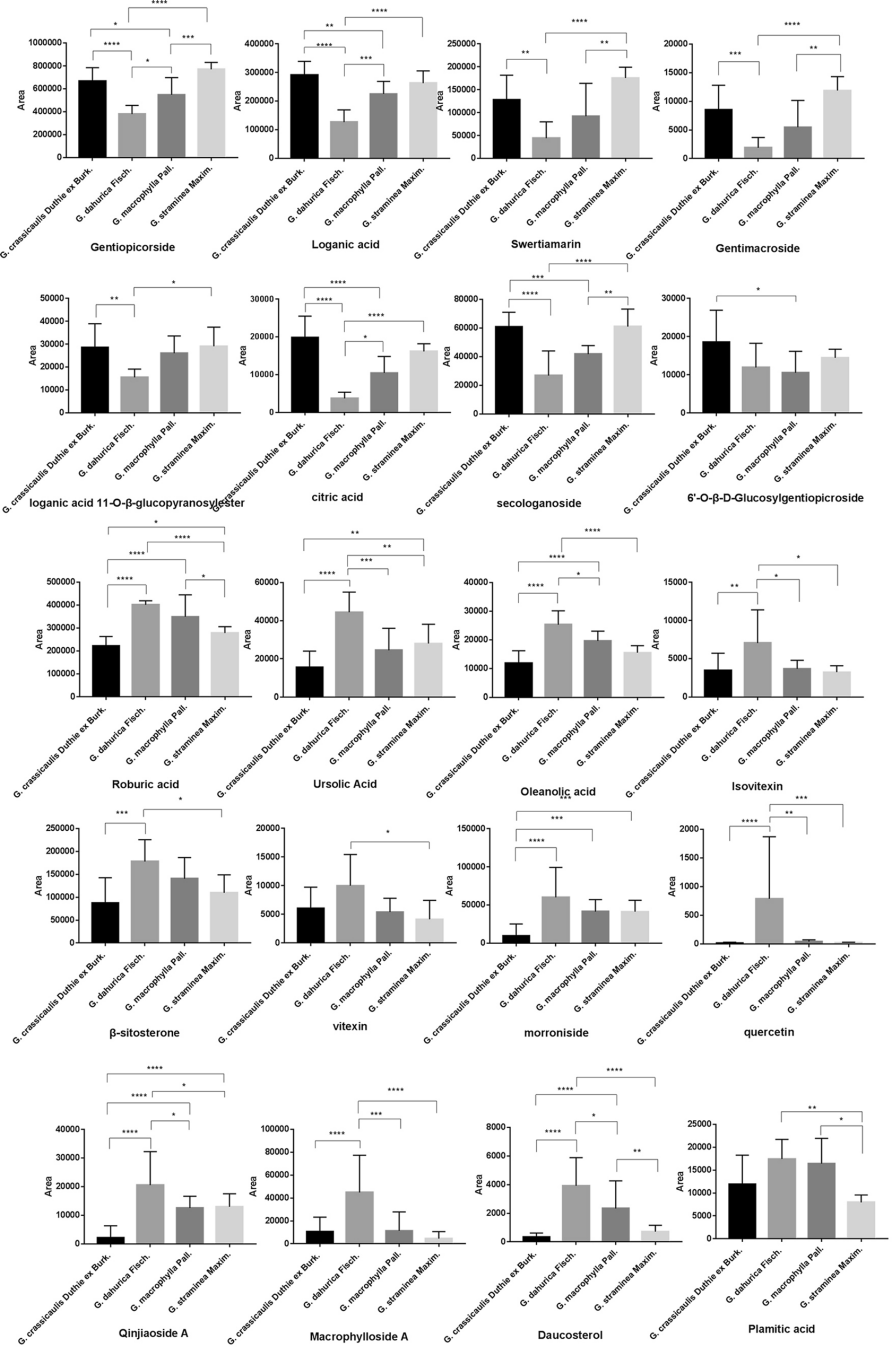
Part of ANOVA test result of identified constituents by TOF–MS. *P < 0.05; **P < 0.01; ***P < 0.001;****P < 0.0001

Representative base peak intensity (BPI) chromatograms of *G. crassicaulis* is shown in Fig. 3. By comparing actual mass, MS/MS fragments and retention time (RT) of target compounds, 28 constituents were identified, among which 17 were further identified by reference standards. The compound information is list in Table 3, with XIC chromatography shown in Additional file 2: Fig. S1. ANOVA followed by Tukey’s multiple comparison test were conducted for these 28 compounds (partly shown in Fig. 4).

**Table 3:** Characterization of chemical constituents in Qinjiao by UHPLC-QTOF–MS in ESI-mode

No.	tR/min	Observed mass	Molecular weight	Molecular formula	MS/MS fragments ions (m/z)	Identified compound
1^a^	0.87	341.1086	342.1161	C_12_H_22_O_11_	179.0562, 161.0459, 131.0351, 119.0350, 89.0241, 59.0135	Sucrose
2^b^	0.88	387.1148	388.1369	C_17_H_24_O_10_	341.1090, 195.0507,179.0562, 59.0238, 59.0136	Swertiajaposide A
3^*^	1.63	191.0196	192.0270	C_6_H_8_O_7_	111.0086, 87.0087, 67.0188, 57.0345	Citric acid
4^b^	2.56	169.0870	170.0943	C_9_H_14_O_3_	123.0806, 121.0663, 110.9975, 95.0503, 67.0559, 61.9881	Isoboonein
5^b^	2.73	315.0719	316.0794	C_13_H_16_O_9_	315.0723, 153.0181, 109.0293	5-(β-d-Glucopyranosyl)-2-hydroxybenzoic acid
6^b^	2.99	537.1828	538.1897	C_22_H_34_O_15_	375.1297,221.0264, 213.0769, 169.0869, 113.0241, 179.0557, 89.0242, 69.0344	Loganic acid 11-*O*-β-glucopyranosylester
7^a^	3.31	399.1258,	376.1369	C_16_H_24_O_10_	213.0766, 169.0868, 151.0761, 119.3047, 113.0240, 89.0241, 69.0342, 59.0135	Loganic acid
8^a^	3.39	451.1446	406.1475	C_17_H_26_O_11_	405.1165, 243.0872, 141.0553, 101.0242, 89.0241, 59.0135	Morroniside
9^a^	3.42	563.1620	518.1635	C_22_H_30_O_14_	221.0659, 179.0552, 161.0449, 119.0343, 89.0237	6′-*O*-β-d-Glucosylgentiopicroside
10^b^	4.49	403.1245	404.1319	C_17_H_24_O_11_	151.0764, 125.0242, 113.0243, 89.0243, 59.0137	Qinjiaoside A
11^b^	3.67	519.1726	520.1791	C_22_H_32_O_14_	519.1743, 323.0995, 213.0767, 151.0762, 125.0244, 59.0142	Swertiapunimarin;6′-*O*-Glucopyranosylsweroside
12^a^	3.73	419.1193	374.1212	C_16_H_22_O_10_	149.0605, 141.0188, 119.0347, 89.0238	Swertiamarin
13^b^	3.89	389.1085	390.1161	C_16_H_22_O_11_	183.0660, 165.0557, 121.0655, 89.0241, 69.0343	Secologanoside
14^a^	4.11	401.1074	356.1107	C_16_H_20_O_9_	149.0604, 119.0347, 89.0239, 71.0136, 59.0133	Gentiopicorside
15^b^	4.17	531.1529	532.1581	C_26_H_28_O_12_	235.0612, 191.0714, 173.0608, 163.0767, 149.0606, 89.0243	Gentimacroside
16^a^	4.4	403.1244	358.1263	C_16_H_22_O_9_	357.1193, 195.0666, 179.0564, 125.0241, 119.0347, 151.0763, 89.0242, 81.0343, 59.0136 9	Sweroside
17^a^	5.78	447.0936	448.1005	C_21_H_20_O_11_	429.1045, 369.0630, 357.0616, 327.0508, 297.0398, 285.0404, 61.9884	Isoorientin
18^a^	6.62	431.0983	432.1056	C_21_H_20_O_10_	341.0668, 323.0563, 311.0559, 283.0608, 281.0451	Isovitexin
19^b^	6.76	477.1032	432.1056	C_21_H_20_O_10_	431.0983, 323.0766, 315.0720, 161.0238, 153.0187, 152.0048	Vitexin
20^b^	7.04	875.2236	876.2324	C_40_H_44_O_22_	875.2251, 833.2133, 739.2096, 713.1740, 577.1562, 535.1466, 315.0723, 153.0191,	Macrophylloside A
21^a^	7.08	301.0345	302.0426	C_15_H_10_O_7_	193.0142, 149.0248, 121.0312	Quercetin
22^a^	7.19	285.0397	286.0477	C_15_H_10_O_6_	192.0061, 177.0194, 142.9487, 119.0135, 87.0237	Kaempferol
23^a^	9.89	455.3531	456.3604	C_30_H_48_O_3_	409.2535, 152.9962	Ursolic acid
24^a^	9.92	455.3529	456.3604	C_30_H_48_O_3_	409.2535, 152.9962	Oleanolic acid
25^b^	10.73	255.2308	256.2402	C_16_H_32_O_2_	255.2326, 237.2210	Plamitic acid
26^a^	13.5	621.4381	576.4390	C_35_H_60_O_6_	575.4681, 303.8955, 295.2278, 191.9469, 89.0241	Daucosterol
27^a^	14.5	457.3658	414.3862	C_29_H_50_O	457.3671, 411.3623	β-Sitosterone
28^a^	15.3	439.3581	440.3654	C_30_H_48_O_2_	439.3565, 421.3469	Roburic acid

^a^Identified by comparing with the reference standards

^b^Putative identifications by MS and MS2 fragmentations

**Fig. 5:**
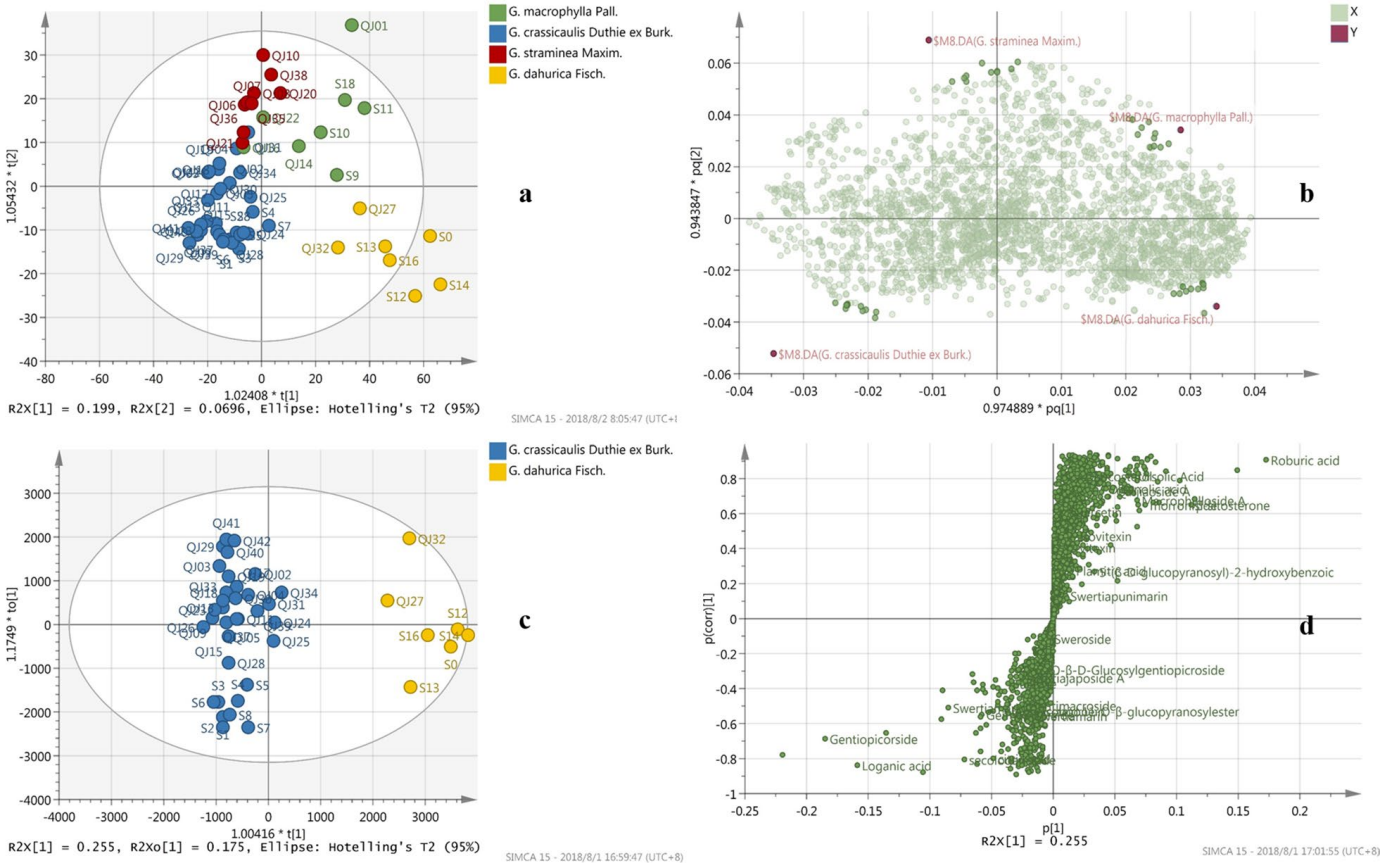
Score plot and loading plot of TOF–MS data. **a** Score plot of 4 species of Qinjiao; **b** loading plot of OPLS-DA model; **c** OPLS-DA score plot of *G. crassicaulis* and *G. dahurica* species; **d** S-plot of *G. crassicaulis* and *G. dahurica* species

Multivariate analysis of the TOF–MS data was carried out. Initially, unsupervised PCA-X analysis were conducted among groups, showing preferably discriminative distribution (not shown, R2X = 0.661, Q2 = 0.398). Subsequently, to maximize the variation among groups and to determine the variables that contributed to this variation, supervised OPLS-DA model (Fig. 5a) was employed among four species of Qinjiao, with R2X = 0.38, R2Y = 0.666, Q2 = 0.549. The loading plot (Fig. 5b) revealed the correlations between class (species) and variables (MASS feature), where variables clustering close to each class were considered to make great contributions to the classification. Furthermore, it was noticed that *G. crassicaulis* and *G. dahurica* clustered furthest from each other in the score plot (Fig. 5a). To explore the difference, OPLS-DA analysis for these two groups was conducted, with R2Y 0.924 and Q2 0.864 (Fig. 5c). Corresponding S-plot (Fig. 5d) was analyzed, which was commonly used and effectively showed the difference between groups. Part of the variable selected from S-plot with VIP value > 1.0 and their abundances were shown in Additional file 3: Table S2. The established OPLS-DA models were further validated by CV-ANOVA test, with P valve less than 0.05 indicating significant models (Additional file 4: Table S3).

## ^1^H-NMR data metabolomics

**Fig. 6:**
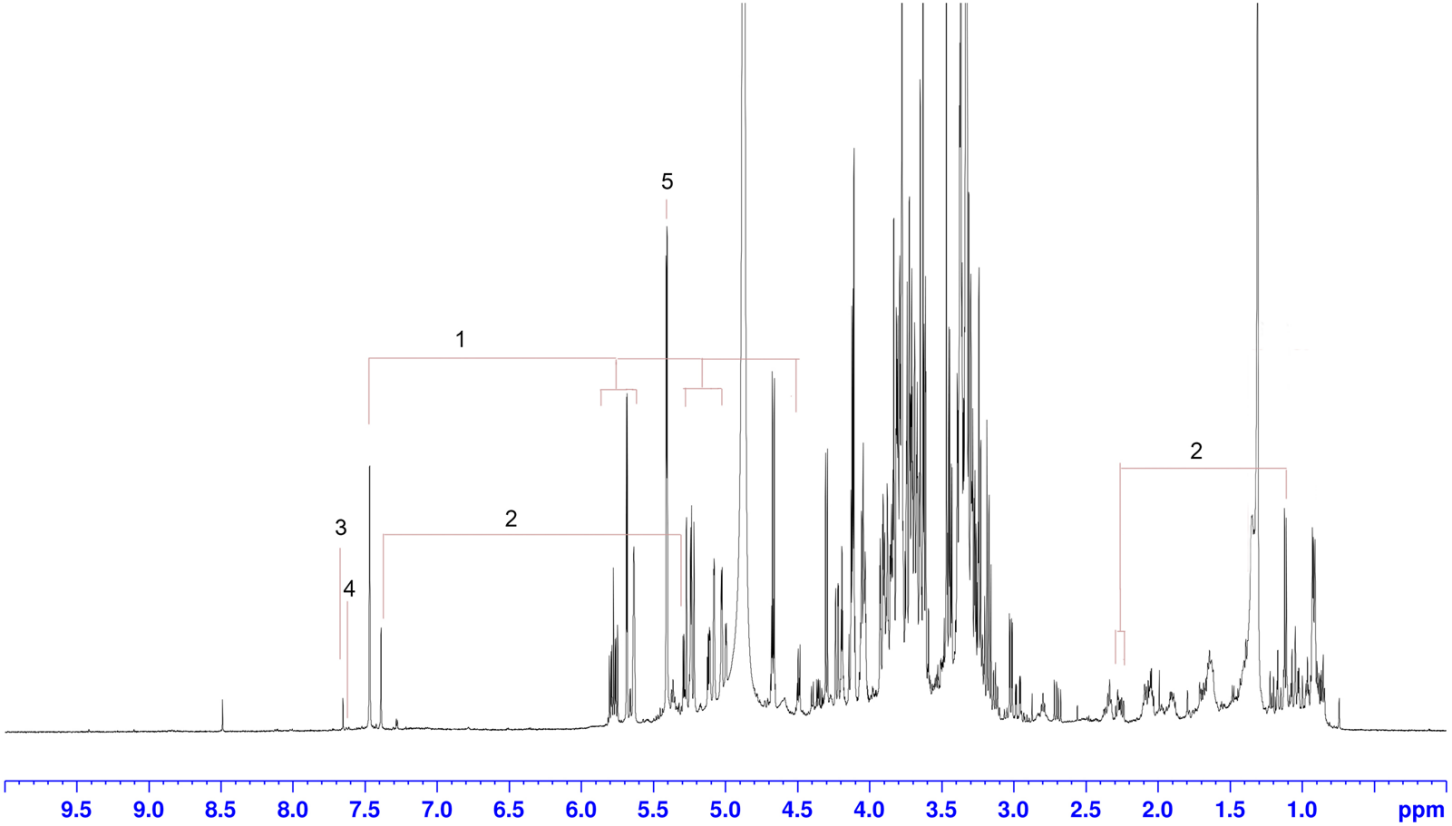
Representative ^1^H-NMR spectrum of *G. crassicaulis* and signal identified. 1. Gentiopicroside, 2. Loganic acid, 3. Swertiamarin, 4. Sweroside, 5. Sucrose

A representative 1D ^1^H-NMR spectrum of G. *crassicaulis* is shown in Fig. 6. Signal assignments were list in Table 4. Fore iridoids, gentiopiciroside, swertiamarin, loganic acid, and sweroside were identified ultimately by Chenomx NMR suit (version7.6, Chenomx, Edmonton, Canada).

**Table 4:** Characteristics of ^1^H-NMR signals observed in Qinjiao extract

Compound	Group	Chemical shift δ (ppm)
Gentiopicroside	3-H(d)	7.47
	8-H(m)	5.76
	1-H(d)	5.66
	6-H(m)	5.63
	10-CH_2_(dt)	5.22
	7=CH(m)	5.00–5.10
	1-glc-1′(d) –CH (anomeric)	4.67
Loganic acid	3-H(d)	7.38
	1-H(d)	5.28
	5-H(m)	2.24
	9-H(m)	1.98
	8-H(d)	1.10
	1-glc-1′(d) –CH (anomeric)	4.66
Swertiamarin	3-H(s)	7.65
	9-H(d)	2.93
Sweroside	3-H(d)	7.60
Sucrose	–CH (anomeric)(d)	5.40

a Letters in parentheses indicate the peak multiplicity property of proton NMR signal

*s* singlet,* d* doublet,* dd* double doublet,* dt* double triplet,* t* triplet,* m* multiplet,* br* broad

**Fig. 7:**
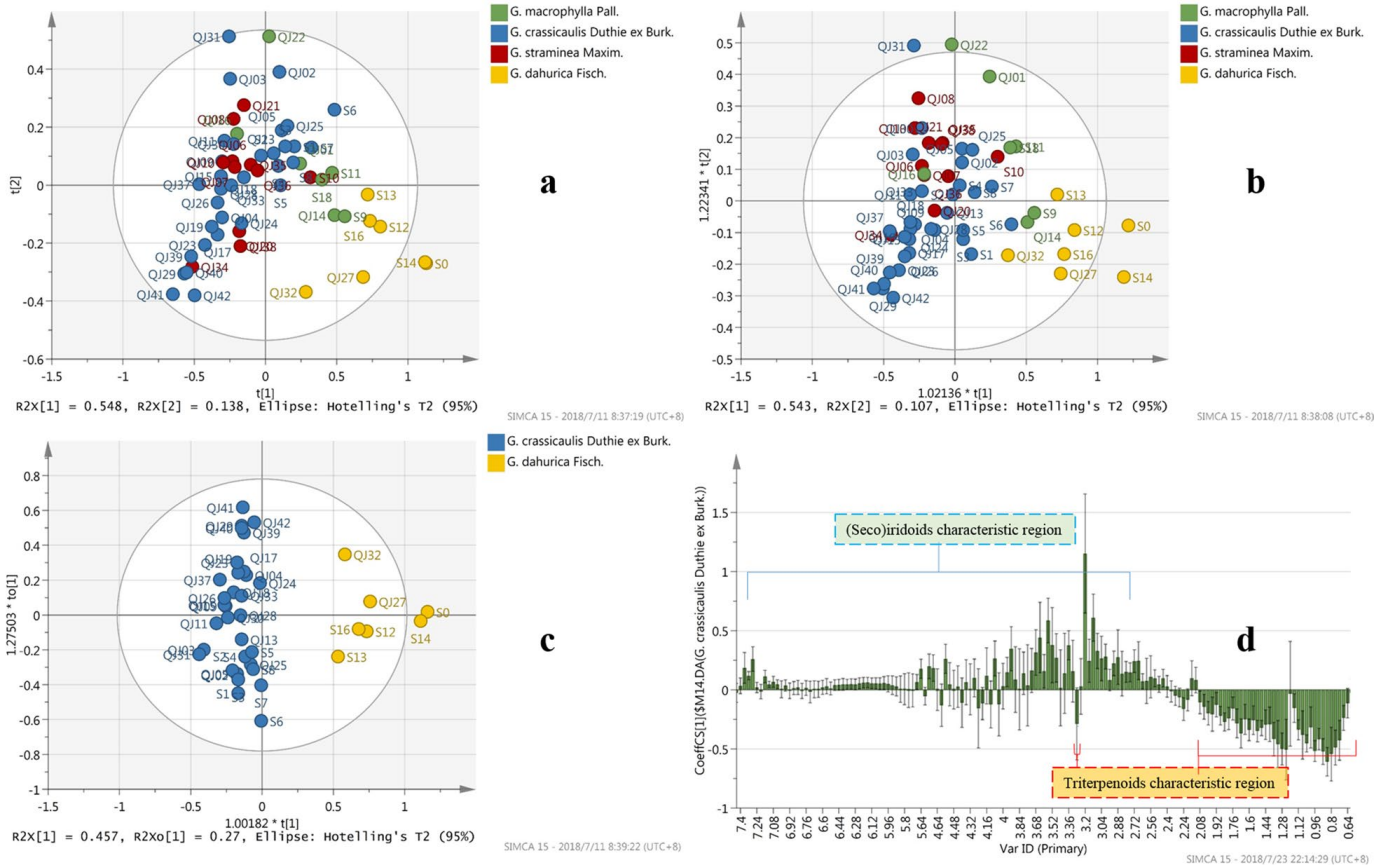
Score plot and loading plot of ^1^H-NMR data. **a**, **b**: PCA and OPLS-DA score plot of 4 species of Qinjiao; **c**, **d**: Score plot and s-plot of OPLS-DA analysis between *G. crassicaulis* and *G. dahurica* species

NMR data acquired was also analyzed by PCA-X model (R2X = 0.943, Q2 = 0.817) and OPLS-DA models (R2X = 0.38, R2Y = 0.666, Q2 = 0.549). As shown in Fig. 7a, b, samples from species of *G. crassicaulis*, *G. straminea*, *G. macrophylla*, and *G. dahurica* distributed regionally in the score plot, with the latter 3 species gradually deviating far away from the first one.

To explore the diversity between *G. crassicaulis* and *G. dahurica*, OPLS-DA analysis was achieved between them, with R2X 0.726, R2Y 0.884 and Q2 0.848 (Fig. 7c). Corresponding S plot (Fig. 7d) was generated. The established OPLS-DA models were further validated by CV-ANOVA test, with P valve less than 0.05 indicating significant models (Additional file 4: Table S3).

## Discussion

### ITS2 data analysis

Minimum distance within species was observed between *G. macrophylla*_and *G. straminea*, with K2P distance value 0.0146. While maximum K2P distance within species was present between *G. crassicaulis* and *G. dahurica*, with value 0.0371. The minimum interspecific distance of ITS2 region was higher than the maximum intraspecific distance, indicating that the ITS2 barcode performed well in the discrimination of four species of Qinjiao. The distances among species revealed were in accordance with discrepancy detected by following metabolomics analysis. It was illustrated that species of *G. macrophylla*, *G. straminea*, *G. dahurica* and *G. crassicaulis* can be clearly distinguished by the NJ tree. ITS2 analysis confirmed the potential discrepancy among species and guaranteed reliability of subsequent metabolomics-based species evaluation.

### TOF–MS data analysis

The score plots (Fig. 5a) shows that samples from species of *G. macrophylla*, *G. straminea*, *G. dahurica* and *G. crassicaulis* located different areas in score plot, inferring distinctive chemical profiles of four species of Qinjiao. In general, *G. crassicaulis* and *G. straminea* were closest in the score plot, while *G. macrophylla* and *G. dahurica* were progressively far away from them. Maximum spatial distance was present between *G. dahurica* and *G. crassicaulis*. The result was consistent with discrepancy revealed by ITS2 analysis. The loading plot (Fig. 5b) revealed MASS features making great contributions to the classification, which was further confirmed by ANOVA test. Flavonoids (isovitexin, morronside, quercetin) and triterpenoids (oleanolic acid, ursolic acid, roburic acid, β-sitosterone, daucosterol), as well as certain iridoids (macrophylloside A and Qinjiaoside A) were significant higher in *G. dahurica*. than other 3 species; while other(seco)iridoids (swertiamarin, secologanoside, loganic acid, gentimacroside, loganic acid 11-*O*-β-glucopyranosylester) and citric aicd were richer in other 3 species of Qinjiao than *G. daurica*. No obvious distinction was detected in contents of sweroside, sucrose, swertiapunimarin, and swertiajaposide A among four species of Qinjiao.

OPLS-DA score plot (Fig. 5c) and corresponding S-plot (Fig. 5d) of *G. crassicaulis* and *G. dahurica* confirmed maximum K2P distance revealed by ITS2 analysis. It was noticed that MASS features of 401.1074_4.19 (gentiopicroside), 375.1276_3.29 (loganic aicd), 419.1193_3.81(swertiamarin), 191.0200_1.09(citric acid), 563.1601_3.50(6′-*O*-β-d-glucosyl-gentiopicroside), and 389.1087_3.89 (secologanic acid) were richer in *G. crassicaulis*, while MASS features of 439.3562_15.30 (roburic aicd), 455.3510_9.93 (ursolic Acid), 455.3505_9.99 (oleanolic acid), 457.3658_14.5(β-sitosterone) and 451.1456_3.40 (morroniside) were richer in *G. dahurica*. The discrepancy was consistent with ANOVA test result (Fig. 4). It seems that (seco)iridoids like loganic acid, gentiopicroside or swertiamarin were richer in specie of *G. crassicaulis*, while flavonoid (morroniside) and triterpenoids (roburic aicd, ursolic acid, oleanolic acid, β-sitosterone) were richer in specie of *G. dahurica*. The discrepancy was consistent with previous reports [19, 20], which reported higher contents of gentiopicroside, loganic acid, sertiamarin, and 6′-*O*-*β*-d-glucosyl-gentiopicroside in *G. crassicaulis* comparing with *G. dahurica*.

## ^1^H-NMR data analysis

According to the score plot (Fig. 7a, c), NMR based metabolomics validated results revealed by MS based metabolomics. The S-plot (Fig. 7d) between these two groups showed that signal abundance from 3 to 7 ppm was richer in *G. crassicaulis*, while which from around 0.8–2 ppm was richer in *G. dahurica*. Unfortunately, limited to the complexity of the ^1^H-NMR spectra, compounds related to the abundance difference were not directly identified. However, the difference was confirmed and can be explained by preceding MS based metabolomics. The signal intensity discrepancy present in ^1^H-NMR spectra coincided with abundance discrepancy MS revealed (Fig. 5d). Richer abundance of triterpenoids in *G. dahurica* produced richer signal intensity in characteristic region range of 0.5 to 2.4 ppm (signal from Skeleton proton) and 3.24 to 3.28 ppm(H-3) [21]. On the other side, higher concentration of (seco)iridoids produced higher signal intensity of 2.4 to 5 ppm (signal from (seco)iridoids skeleton proton) and 7.0–7.4 ppm(signal of H-3) [22]. The consistence by NMR and TOF–MS metabolomics confirmed the chemical discrepancy between *G. crassicaulis* and *G. dahurica*. The discrepancy may be helpful for distinguishing of these two species and may lead to potential pharmacodynamics discrepancy, which remains to be investigated.

### ITS2, TOF–MS and NMR integration

ITS2 based gene comparison can present species distance or genetic relationship among species. Currently, ITS2 is the mostly commonly used region for the barcoding and authentication of herbal medicinal materials. In future, species authentication by ITS2 sequence (or other genetic method) may be precondition of quality evaluation of MSRHM. However, environmental implication and chemical discrepancy were not reflected. Information of quality and origin shall ultimately rely on chemical method. On the other side, TOF–MS or NMR based metabolomics analysis providing systemic chemical information among species, and can be powerful tool for quality investigations. TOF–MS bears the advantage of high sensitivity, high specificity, and thus offers maximum amount of information for quality control. However, TOF–MS based platform suffers from the disadvantage of inhomogeneous ionization propensities and time-consuming samples preparation procedures. In contrast, NMR platform possesses disadvantages of lower sensitivity and limited specificity, as well as advantages of homogenous signal response and simple sample preparations [23]. In general, a combination of ITS2 sequence comparations and TOF–MS as well as NMR based metabolomics analysis can validated each other and provided more comprehensive quality investigation for MSRHM [24].

## Conclusion

In this study, a new integrated quality evaluation strategy was proposed for MSRHM of Qinjiao employing ITS2 sequence comparation, TOF–MS and NMR based metabolomics analysis. At first, gene comparison based on ITS2 sequence was conducted among 4 species of Qinijao. Then, TOF–MS and NMR based metabolic analysis were applied to investigate species discrepancy among four species of Qinjiao. It turned out that species discrepancy revealed among species were consistent by ITS2 sequencing, NMR and TOF–MS based metabolomics. Maximum species difference was noticed between *G. crassicaulis* and *G. dahurica*. Chemical difference among species based on TOF–MS and NMR were tentative explored. For TOF–MS profiling of Qinjiao, 28 constituents were tentative identified, 17 of which were further confirmed by standards. For ^1^H-NMR spectra of Qinjiao, signals from 5 compounds were assigned. Contents discrepancies were investigated by ANOVA analysis. It turned out that MS based metabolomics coincided with NMR based metabolomics result, and explained the intensity discrepancy in ^1^H-NMR spectra.

The current research demonstrates that integration of ITS2 sequence comparation and UPLC/Q-TOF MS as well as ^1^H-NMR based metabolomics analysis can be a powerful strategy for quality investigation of MSRHM.

## Supplementary information

**Additional file 1: Table S1.** Drift of retention times, *m/z* and the RSD of peak areas of 5 selected characteristic features from QC samples during the analysis.

**Additional file 2: Fig. S1.** XIC chromatography of 28 identified constituents.

**Additional file 3: Table S2.** Part of the variable with VIP value > 1.0 from OPLS-DA analysis of *G. crassicaulis* and *G. dahurica.*

**Additional file 4: TAble S3.** P value of CV-ANOVA for OPLS-DA models based on MS or NMR analysis.

## Data Availability

The datasets used and/or analyzed during the current study are available from the corresponding author on reasonable request.

## Abbreviations

MSRHM: multi-species resourced herb medicine
*G. macrophylla*: *Gentiana macrophylla* Pall
*G. straminea*: *Gentiana straminea* Maxim
*G. crasicaulis*: *Gentiana crassicaulis* Duthie ex Burk
*G. daurica*: *Gentiana dahurica* Fisch
ITS2: internal transcribed spacer 2
UPLC-Q-TOF–MS: ultra performance liquid chromatography quadrupole time-of-flight mass spectrometry
^1^H-NMR: proton nuclear magnetic resonance spectrometer
PCA: principal component analysis
OPLS-DA: orthogonal partial least squares discrimination analysis
ANOVA: one-way analysis of variance
VIP: variable importance in the projection value
