# Supplementary material for: Integrated quality evaluation strategy for multi-species resourced herb medicine of Qinjiao by metabolomics analysis and genetic comparation
Source: Chin Med. 2020 Feb 11;15:16. doi: 10.1186/s13020-020-0292-3 (PMC7014644; doi:10.1186/s13020-020-0292-3)
Supplement: Supplementary file 1 — Additional file 1: Table S1. Drift of retention times, m/z and the RSD of peak areas of 5 selected characteristic features from QC samples during the analysis. [file 13020_2020_292_MOESM1_ESM.docx]

Table S1 Drift of retention times, m/z and the RSD of peak areas of 5 selected characteristic features from QC samples during the analysis.

| Mass features | Drift of RT(min) | Drift of MS (PPB) | RSD of peak areas |
| --- | --- | --- | --- |
| 0.879_241.1089 | 0.021 | 3.84 | 5.73 |
| 3.313_751.2657 | 0.027 | 2.93 | 3.77 |
| 4.199_401.1084 | 0.033 | 2.74 | 3.08 |
| 8.152_717.4595 | 0.031 | 4.32 | 4.43 |
| 13.409_457.3684 | 0.032 | 2.62 | 6.05 |
