# Supplementary material for: Integrated quality evaluation strategy for multi-species resourced herb medicine of Qinjiao by metabolomics analysis and genetic comparation
Source: Chin Med. 2020 Feb 11;15:16. doi: 10.1186/s13020-020-0292-3 (PMC7014644; doi:10.1186/s13020-020-0292-3)
Supplement: Supplementary file 3 — Additional file 3: Table S2. Part of the variable with VIP value > 1.0 from OPLS-DA analysis of G. crassicaulis and G. dahurica. [file 13020_2020_292_MOESM3_ESM.docx]

Table S2 Part of the variable with VIP value>1.0 from OPLS-DA analysis of *G. crassicaulis* and *G. dahurica*.

| Var ID (Primary) | Var ID (Var. Sec. ID:1) | VIP |
| --- | --- | --- |
| 401.1074_4.19_group_(Monoisotopic) | Gentiopicorside | 9.94 |
| 439.3562_15.30_group_(Monoisotopic) | Roburic acid | 8.13 |
| 375.1276_3.29_group_(Monoisotopic) | Loganic acid | 7.50 |
| 457.3658_14.50_group_(Monoisotopic) | Β-sitosterone | 5.52 |
| 419.1193_3.81_group_(Monoisotopic) | Swertiamarin | 5.34 |
| 451.1456_3.40_group_(Monoisotopic) | Morroniside | 3.64 |
| 355.1025_4.17_group_(Monoisotopic) | Gentiopicorside | 3.51 |
| 389.1087_3.89_group_(Monoisotopic) | Secologanoside | 3.44 |
| 875.2236_7.04_group_(Monoisotopic) | Macrophylloside A | 3.21 |
| 455.3510_9.93_group_(Monoisotopic) | Ursolic acid | 3.09 |
| 191.0184_1.62_group_(Monoisotopic) | Citric acid | 2.31 |
| 449.1279_3.54_group_(Monoisotopic) | Qinjiaoside A | 2.28 |
| 455.3505_9.99_group_(Monoisotopic) | Oleanolic acid | 2.01 |
| 341.1083_0.87_group_(Monoisotopic) | Sucrose | 1.87 |
| 537.1818_2.99_group_(Monoisotopic) | Loganic acid 11-O-β-glucopyranosylester | 1.79 |
| 315.0700_2.73_group_(Monoisotopic) | 5-(β-D-glucopyranosyl)-2-hydroxybenzoic acid | 1.56 |
| 517.1570_3.50_group_(Monoisotopic) | 6'-O-β-D-Glucosylgentiopicroside | 1.55 |
| 565.1771_3.67_group_(Monoisotopic) | Swertiapunimarin | 1.50 |
| 531.1529_4.17_group_(Monoisotopic) | Gentimacroside | 1.48 |
| 255.2308_10.73_group_(Monoisotopic) | Plamitic acid | 1.47 |
| 387.1137_0.87_group_(Monoisotopic) | Swertiajaposide A | 1.38 |
| 373.1129_3.80 | Swertiamarin | 1.13 |
| 575.4665_13.57_group_(Monoisotopic) | Daucosterol | 1.05 |
