# Supplementary material for: Integrated quality evaluation strategy for multi-species resourced herb medicine of Qinjiao by metabolomics analysis and genetic comparation
Source: Chin Med. 2020 Feb 11;15:16. doi: 10.1186/s13020-020-0292-3 (PMC7014644; doi:10.1186/s13020-020-0292-3)
Supplement: Supplementary file 4 — Additional file 4: TAble S3. P value of CV-ANOVA for OPLS-DA models based on MS or NMR analysis. [file 13020_2020_292_MOESM4_ESM.docx]

Table S3 P value of CV-ANOVA for OPLS-DA models based on MS or NMR analysis

| OPLS-DA models | P value of CV-ANOVA (p-value<0.05 as pointing to a significant model) |
| --- | --- |
| MS based OPLS-DA of 4 kinds of Qinjiao | 4.25771e-11 |
| MS based OPLS-DA of *G. crassicaulis* and *G. dahurica* species | 0.0239163 |
| NMR based OPLS-DA of 4 kinds of Qinjiao | 0.0189825 |
| NMR based OPLS-DA of *G. crassicaulis* and *G. dahurica* species | 6.55084e-14 |
